# Facile fabrication, characterization and catalytic activity of a NiMo/Al_2_O_3_ nanocatalyst *via* a solution combustion method used in a low temperature hydrodesulfurization process: the effect of fuel to oxidant ratio†

**DOI:** 10.1039/d0ra01590c

**Published:** 2020-03-26

**Authors:** Roya Hamidi, Reza Khoshbin, Ramin Karimzadeh

**Affiliations:** Chemical Engineering Faculty, Tarbiat Modares University P. O. Box 14115-114 Tehran Islamic Republic of Iran ramin@modares.ac.ir +98-21-88006544 +98-21-82883315; Department of Chemical and Materials Engineering, Buein Zahra Technical University Buein Zahra Qazvin Iran

## Abstract

Novel bimetallic NiMo/Al_2_O_3_ nanocatalysts were fabricated *via* a solution combustion method to evaluate the role of fuel to oxidant molar ratios on their structural properties and hydrodesulfurization activity. The citric acid/oxidant ratios of 0.5, 1, 2 and 4 were selected to address the optimum ratio. Characterization results demonstrated that the content of citric acid considerably influenced the morphological and textural properties of the nanocatalysts. Such morphology modification is attributed to the consequent difference of the effluent exhaust gas during combustion. We show that with our method a relatively homogeneous distribution of the active material over the support can be achieved. The obtained data from N_2_ adsorption–desorption analysis illustrated that at a fuel/oxidant ratio of 4 the external and surface area were *ca.* 2.1 and 1.5 times more than the corresponding one in the fuel/oxidant ratio of 0.5, respectively. Furthermore, a higher amount of fuel can improve the catalyst reducibility by decreasing the interaction of metal active phase with the support surface. The catalytic performance of sulfided nanocatalysts is evaluated in a slurry reactor, operated at ambient pressure using high thiophene contamination as a model fuel. The solution combustion synthesis method was able to remove 100% of the sulfur compound in the reaction medium.

## Abstract 2

Novel bimetallic NiMo/Al_2_O_3_ nanocatalysts were fabricated *via* a solution combustion method to evaluate the role of fuel to oxidant molar ratios on their structural properties and hydrodesulfurization activity.

## Graphical abstract

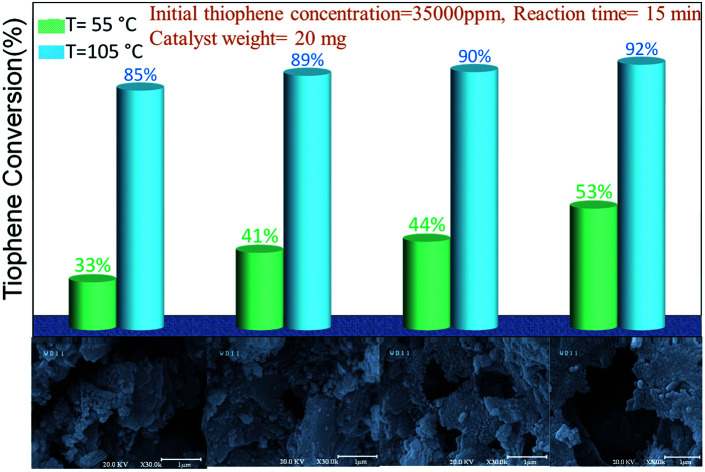

## 1 Introduction

Refined petroleum products such as gasoline and diesel fuels usually contain a significant amount of sulfur compounds which produce SO_*x*_ gases. These gases reduce the efficiency of the petrochemical units and cause corrosion to the refinery equipment.^1,2^ Also, such emission may cause health problems when inhaled, and in the environment, it forms acid rain which can have negative effects on ecosystems. Because of these unpleasant consequences, environmental regulation has been increased aiming at intense reduction of the sulfur content level to 10 μg g^−1^.^3,4^ Among all the physiochemical methods employed for desulfurization, such as adsorptive desulfurization, hydrodesulfurization, supercritical water desulfurization and oxidative desulfurization, the hydrodesulfurization (HDS) process has received more attention for removing organic sulfur. This catalytic reaction is generally carried out at high temperature and hydrogen partial pressure.^5,6^

Generally, hydrotreating reactions are catalyzed by molybdenum/tungsten disulfide and promoted with cobalt/nickel, which is placed at the edge of MoS_2_/WS_2_ slabs.^10–12^ Numerous substances have been applied as support in the HDS process, including gamma-alumina, zeolites, metal oxides, carbon-based material, silica and their composite.^13–16^ Among of them, gamma-alumina has considered the most commonly used because of low cost, moderate acidsites, considerable mechanical properties and adequate porosity.^17,18^

In order to obtain superior hydrodesulfurization performance, modified catalyst should be utilized, which could be achieved by the employment of novel preparation methods or incorporation of suitable promotors. Positive effects of various promotors such as zirconia,^18^ titania,^19^ fluorine,^20^ boron^21^ and phosphorus^22^ have been reported by numerous studies. It is also well established that the addition of chelating ligands in the catalyst preparation is a beneficial way to improve catalytic performance in the HDS process.^23^ In the presence of chelating agents, molybdate and tungstate anions are not strongly bonded to the support. This issue makes them easily sulfided to MoS_2_ and WS_2_ crystallites which are the catalytically active sites.^24^

In literature, impregnation method attracted widespread attention for the preparation of HDS catalysts. However, several methods such as precipitation, sol–gel, chemical vapor deposition, sonochemical and hydrothermal deposition have been applied in the last decades.^16,20,25–29^ Although the above-mentioned preparation methods have had the opportunity to produce advanced hydrodesulfurization catalysts, they suffer from some drawbacks. Some of these disadvantages are relatively complicated equipment, prolonged synthesis time, multistage process, high-energy consumption, poor control of physicochemical properties, need subsequent milling process, expensive starting substance and environmental concerns. These drawbacks make them unsuitable for large scale production.^30,31^ Consequently, an alternative method, called urea matrix combustion, was explored by González-Cortés *et al.*^32–34^ to overcome the aforementioned drawbacks. Their results demonstrated that the generation of mono and polymolybdate with superior reducibility have been facilitated by the urea–organic matrix compared with the impregnation and chelating methods, and accordingly increase the catalyst performance.

This method is an effective time and energy-saving technique for the synthesis of various inorganic powders with high purity and excellent characteristics which is based on a redox reaction between an organic fuel and oxidant.^21^ Solution combustion synthesis (SCS) involves a self-sustained exothermic reaction in a homogeneous mixture of different oxidants, including nitrate, sulfates and carbonates, and fuels such as citric acid, urea, glycine, sucrose, ethylene glycol. The initial solution self-ignites by preheating it to a moderate temperature which leads to the formation of fine solid powders.^35^ The as-combusted material has a porous structure which is designed by the liberation of gaseous by-products like CO_2_, H_2_O, N_2_, CO during the process.^36^ Initial heating in the solution combustion reaction can be applied using a normal oven or irradiating waves such as microwaves.^37^ Manikandan *et al.*^38^ reported Fe_3_O_4_ powders prepared by microwave heating had higher surface area and smaller crystallite size compared to conventional heating. Also, the combustion reaction rate depended on pH values as investigated by Radpour *et al.*^39^ Fuel to oxidant ratio is another parameter due to its important role in the combustion method. In this sense, Zhang *et al.*^40^ studied the role of fuel content on the porous structure of as-combusted Fe_3_O_4_ powders. Briefly, fuel type, fuel to oxidant ratio, heating method and pH value of prepared gel must be carefully adjusted to obtain optimum results in the SCS method.

It is important to say that although the application of the combustion technique has been broadly used for the preparation of various materials for different applications, just limited studies investigated this kind of treatment on the synthesis of HDS catalysts. The aim of the current work was centralized on study the influence of fuel/oxidant molar ratio on the properties of NiMo/γ-A1_2_O_3_ HDS catalysts utilizing citric acid to oxidant molar ratio of 0.5, 1, 2, 4. X-ray diffraction (XRD), thermogravimetric analysis (TGA), field emission scanning electron microscopy (FESEM), energy dispersive X-ray spectrometry (EDX), transmission electron microscopy (TEM), N_2_ adsorption–desorption and temperature-programmed reduction (H_2_-TPR) techniques were conducted to characterize the properties of as-combusted NiMo/Al_2_O_3_ catalysts. The performance of prepared samples has been investigated in HDS reactions using 3.5 wt% thiophene in *n*-decane as model feed operated at atmospheric pressure. Ultimately, the evaluation of thiophene concentration in the treated product was conducted by GC analysis.

## 2 Materials and methods

### 2.1 Materials

Starting substances which used for the synthesis of NiMo supported catalyst were γ-alumina (as support), provided from Ardakan Industrial Ceramic Co. (Yazd, Iran), (NH_4_)_6_Mo_7_O_24_·4H_2_O (as molybdenum source) and Ni(NO_3_)_2_·6H_2_O (as nickel source) supplied by Merck company. Citric acid (Ghatran shimi) and ammonia (Merck) were used for the combustion synthesis method. Also, to assess the HDS performance of catalysts, C_10_H_22_ (Merck) and C_4_H_4_S (Merck) were utilized as solvent and model sulfur compound, respectively. Furthermore, CS_2_ (Merck) and C_7_H_16_ (Merck) were used for sulfiding of NiMo supported catalysts.

### 2.2 Nanocatalyst preparation and procedures

The solution combustion approach was chosen to prepare NiMo/Al_2_O_3_ catalysts with various amounts of fuel to oxidant molar ratio. The synthesis procedure is schematically shown in Fig. 1S in ESI.† First, a separate amount of (NH_4_)_6_Mo_7_O_24_·4H_2_O (22 wt% MoO_3_) and Ni(NO_3_)_2_(H_2_O)_6_ (5 wt% NiO), as Mo and Ni precursors, were dispersed in the minimum volume of distilled water, followed by magnetic stirring to achieve an aqueous solution. Next, γ-alumina powder was mixed with solution and stirred for 60 min at room temperature. In the next stage the fuel was supplied by adding the citric acid to the mixture with fuel/oxidant molar ratios of 0.5, 1, 2, 4. For this reason, 0.42, 0.85, 1.65 and 3.39 g of citric acid was added to the slurry, respectively, which was calculated using eqn (1) and (2).

The solution was kept under stirring at ambient temperature for 1 h. Then the ammonia solution was used under stirring to adjust the pH of the mixture to 7. The slurry was maintained on a hot plate with continuous stirring at about 70 °C to evaporate all excess water to obtain a viscous gel. Afterward, the fuel-based gel was combusted in a crucible which placed in an electric furnace at 550 °C under air atmosphere. The reaction performed in a few minutes. Consequently, fluffy black powder was obtained. Ultimately, to burn off the remaining carbon species, the as-combusted samples were calcined at 550 °C for 4 h. The NiMo/Al_2_O_3_ catalysts are named as NMACA-*x*, where *x* is fuel/oxidant molar ratios of 0.5, 1, 2, 4.

The chemical equation during the combustion process for each of the species by considering H_2_O, CO_2_ and N_2_ as the reaction gases are as follows:

1 Ni(NO_3_)_2_·6H_2_O + 2*φ*C_6_H_8_O_7_·H_2_O + (9*φ* − 5/2)O_2_ → NiO + N_2_ + (10*φ* + 6)H_2_O + 12*φ*CO_2_

2 (NH_4_)_6_Mo_7_O_24_·4H_2_O + 2*φ*C_6_H_8_·H_2_O + (9*φ* + 9/2)O_2_ → 7MoO_3_ + 3N_2_ + (10*φ* + 16)H_2_O + 12*φ*CO_2_

Here, the stoichiometric mixed solution (*φ* = 1) does not need atmospheric oxygen for achieving complete fuel oxidation, while fuel lean or (rich) refers to *φ* < 1 (or >1), respectively.

### 2.3 Nanocatalyst characterizations

Phase identification and structure of catalysts were investigated by XRD analysis using an X'Pert MPD diffractometer (Philips, Netherland) with Co-Kα radiation (*λ* = 0.178897 nm) and input voltage of 40 kV. In order to investigate the morphology of prepared catalysts, FE-SEM analysis was carried out using S4160 (HITACHI, Japan). Surface element composition was detected by energy dispersive X-ray spectrometry (EDX) using a VEGA\\TESCAN-LMU apparatus. The transmission electron microscopy (TEM) analysis was performed by a Philips CM300 device, to evaluate the dispersion of active metals on alumina. To investigate the textural properties of the metal oxide samples, nitrogen adsorption–desorption isotherms data were recorded by the Micrometric (USA) instrument. The surface areas of the obtained samples were calculated by the Brunauer–Emmett–Teller (BET) analysis. Moreover, *t*-plot method was used to estimate the external surface area. To study the thermal decomposition of the dried gels, thermogravimetry analysis was performed using a TGA 209 F3Tarsus instrument. This examination is carried out in air atmosphere from room temperature to 600 °C with a heating rate of 10 °C min^−1^. The reducibility behaviour of supported metal oxide was evaluated by temperature-programmed reduction (H_2_-TPR) using a BELCAT (Japan) instrument.

### 2.4 Experimental apparatus for HDS performance test

Before the hydrodesulfurization process, the prepared catalysts were sulfided by 7 wt% CS_2_-heptane mixture as follows: 2 g catalyst was loaded in the middle of the continuous fixed-bed reactor bounded by two pieces of quartz wool. The sulfidation process was conducted at atmospheric pressure for 3 h at 400 °C under hydrogen (120 ml min^−1^) and nitrogen (50 ml min^−1^) flows.

Catalytic performance toward HDS reactions has been carried out in a slurry reactor using 3.5 wt% thiophene diluted in decane as a sulfur model compound. The HDS reactor configuration is schematically displayed in Fig. 2S in ESI.† The reactor was loaded with 20 mg of sulfided synthesized catalyst and 25 ml decane solution containing the sulfur compound. During the reactions, the hydrogen stream was sparged into the reactor (50 cm^3^ min^−1^). The HDS reaction was conducted at 55 and 105 °C at atmospheric pressure. In order to investigate the effect of residence time on catalytic activity, the experiments were done in two times of 15 and 30 minutes. The concentration of sulfur in the feed and products has been analyzed by a gas chromatography (Teif Gostar Faraz, Iran) equipped with a DB-1 column (Agilent) and flame ionization detector (Agilent). Conversion of thiophene was used to evaluate a catalytic activity in HDS reaction, which was measured by the following equation:
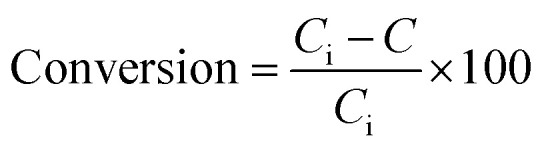
where *C*_i_ is the initial thiophene content (equal to 35 000 ppm) and *C* is the thiophene content in the treated product.

## 3 Results and discussions

### 3.1 Characterization of Ni–Mo/Al_2_O_3_ nanocatalyst

#### 3.1.1 XRD analysis

**Fig. 1:**
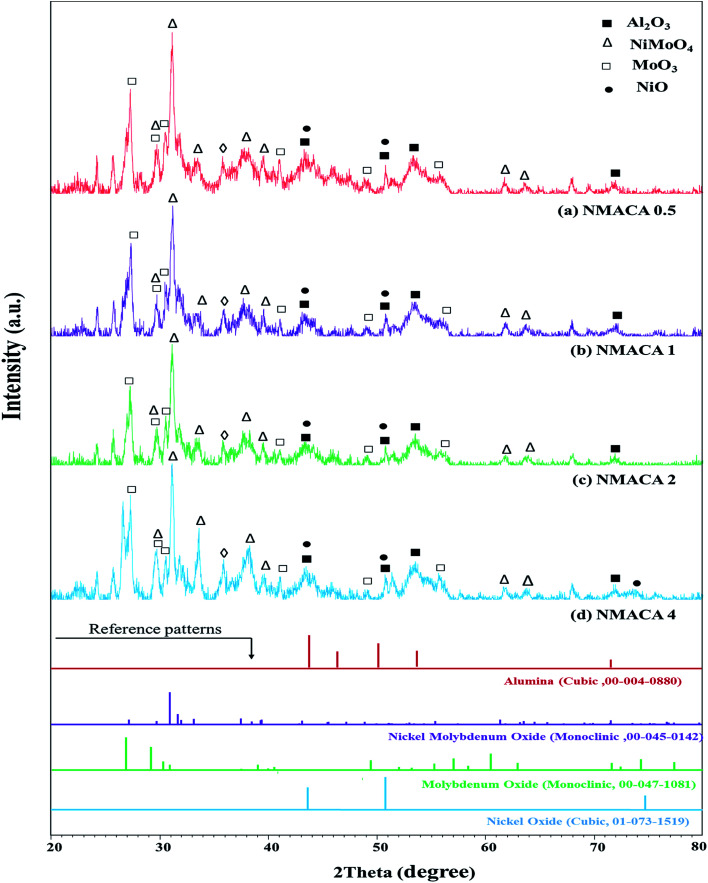
XRD patterns of NiMo/Al_2_O_3_ catalysts with various fuel content (a) NMACA0.5 (b) NMACA1 (c) NMACA2 (d) NMACA4.

The XRD patterns of the prepared NiMo catalysts at different fuel content are illustrated in Fig. 1. Considering the diffraction spectrum depicted, all the peaks at 2*θ* = 43.76, 46.41, 50.1, 53.7, 71.55° assigned to (311), (222), (321), (400) and (511) planes confirm the cubic structure of alumina with the JCPDS no. 00-004-0880.

A series of diffraction peaks at about 26.79, 29.12, 30.24, 30.8, 49.37 and 56.92° could be ascribed to the formation of monoclinic molybdenum oxide (JCPDS 00-047-1081), which was present in the all samples. Furthermore, the reflection peaks at 2*θ* = 27.19, 30.96, 31.98, 33.18, 37.5, 39.38, 61.39 and 63.54° are assigned to the monoclinic crystalline phase NiMoO_4_ (JCPDS no. 00-045-0142). The analysis also reveals that in the case of NMACA4 the intensity of reflection at 31.9° increases, which is the active phase in the HDS reactions.

**Fig. 2:**
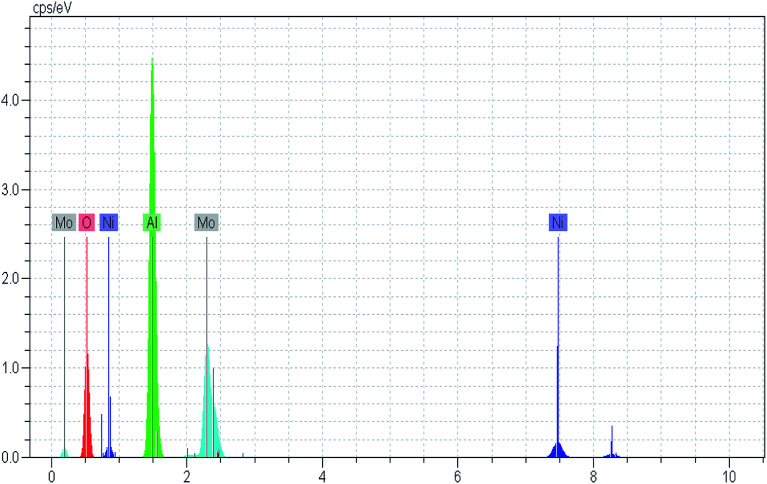
EDX analysis of NiMo/Al_2_O_3_ prepared with combustion synthesis method with fuel/nitrate = 4.

Costa *et al.*,^30^ reported that increasing fuel content leads to prolonged reaction flame time, and subsequently alter the specific characteristics of the obtained products. In this regard, in the case of NMACA4, time of combustion is sufficient to crystallize active phase. It is worth mentioning that, the crystallites bigger than 2–5 nm can be detected by XRD.^4^ The presence of peaks assigned to the NiMoO_4_ and MoO_3_ which are the precursors for the generation of Ni–Mo–S active site, propose the formation of well-crystallized of those phases. This result is in line with former investigation.^4^ In addition, the presence of metal species deposited on the alumina surface in the case of the NMACA4 catalyst was investigated using EDX analysis and the result is shown in Fig. 2. According to this figure, the existence of nickel, molybdenum, oxygen and aluminum species was confirmed.

#### 3.1.2 TGA analysis

**Fig. 3:**
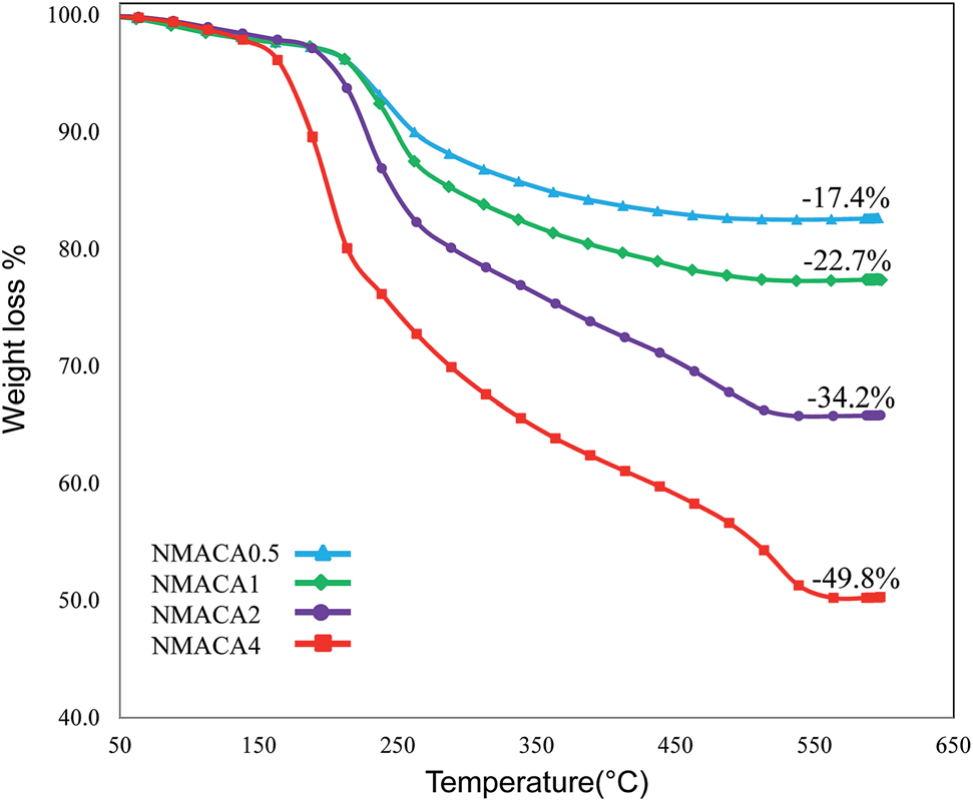
Thermogravimetric analysis of the alumina supported NiMo oxidic precursors.

TGA curves of the not-combusted NiMo/Al_2_O_3_ gels synthesized with different fuel content are plotted in Fig. 3. Looking at this curve, it can be observed that at the 25–600 °C interval all the prepared catalysts demonstrated similar profiles, but with different weight losses.

The weight loss below 200 °C can be assigned to the removal of physically adsorbed moisture.^41^ The sharp decrease in the sample weight above 200 °C is due to different chemical reactions, for example, dehydration–decomposition of deposited Ni and Mo precursors and further combustion of citric acid which are similar to previous reports.^33,42^

Through the combustion process, different successive stages including melting, dissolution and some chemical reactions occur to transform the fuel and the Ni and Mo precursors structure from an amorphous phase to a crystalline phase.^43^ This phenomenon is confirmed by XRD analysis. Furthermore, the combustion type varies from flaming to smoldering as the fuel content increased.^41^ Results from TGA patterns showed that the total weight loss over the NMACA0.5, NMACA1, NMACA2 and NMACA4 is 17.39, 22.69, 34.19 and 49.76 wt%, respectively. Based on the obtained results, amount of weight loss enhanced proportionally with increasing fuel to oxidant ratio. It can be concluded that when higher fuel content was used, more gaseous products was released. Similar results have been obtained by Mendoza-Nieto *et al.*,^5^ They reported that using of citric acid resulted in better dispersion of NiMoW over SBA-15 and led to remarkable improvement in HDS of dibenzothiophenes.

The absence of weight loss above 550 °C indicated that all the organic species the prepared gel would be decomposed with calcination in the air stream at 550 °C. So, that temperature was chosen for the calcination of the synthesized NiMo catalysts.

#### 3.1.3 FESEM analysis

**Fig. 4:**
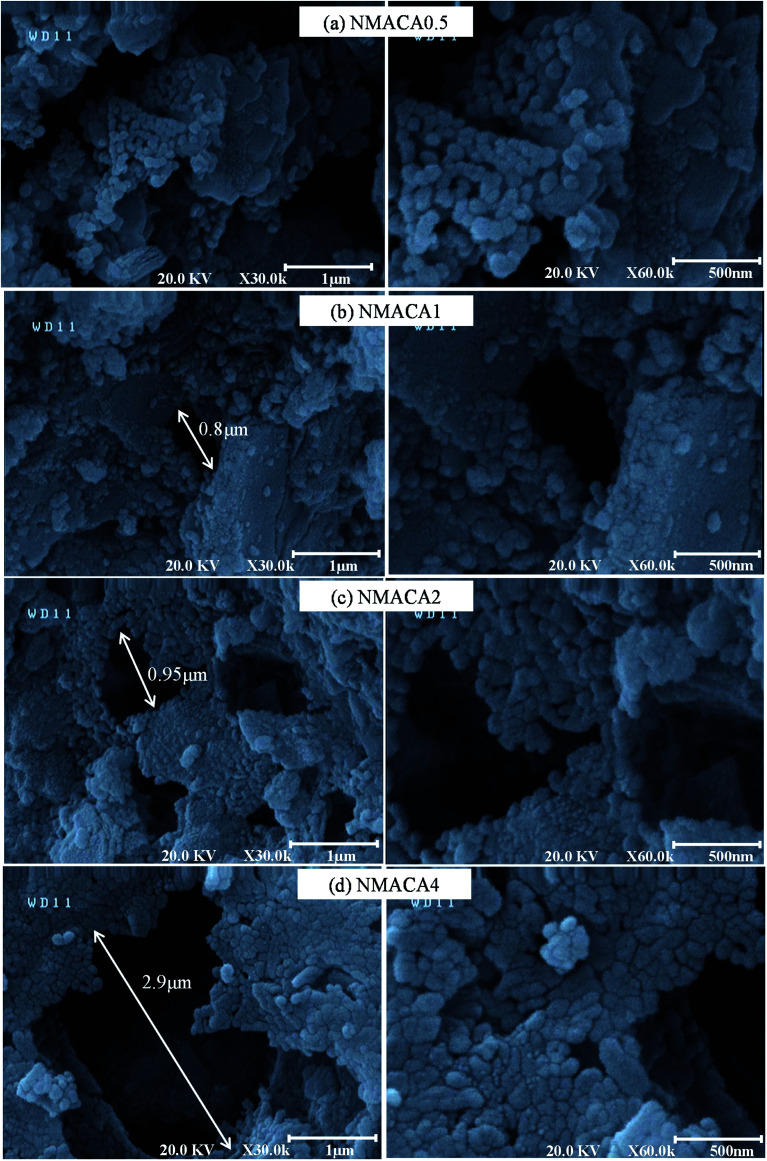
FESEM images of NiMo/Al_2_O_3_ catalysts synthesized with various fuel content (a) NMACA0.5, (b) NMACA1, (c) NMACA2, (d) NMACA4.

The morphologies of the as-combusted nanocatalysts synthesized in different fuel to oxidant molar ratios are shown in Fig. 4. In the case of NMACA0.5, agglomeration of interlinked spherical particles with a particle size around 50 nm have been observed in Fig. 4. While, the powder synthesized at fuel/oxidant ratio of 1 exhibited some surface holes along with agglomerated particles (Fig. 4(b)). After incrementing the fuel to oxidant ratio from 1 to 4, the FESEM images express considerable holes, owing to the escaping of more amounts of gases Fig. 4(c and d). This issue assists the synthesis of porous catalysts by preventing the creation of a agglomerated structure. As seen in Fig. 4, the dimension of combustion pore increased as the fuel content enhanced. The result is consistent with previous findings in literature.^44,45^ In addition, the samples have foamy and sponge-like structure which arise from the intrinsic feature of the combustion synthesis method due to release of large volume of gases.^27^

The above observation revealed that the change in fuel to oxidant ratio has an outstanding effect on the porosity of synthesized catalysts. The highest porous morphology was achieved for the NMACA4 sample. The more gaseous product is released, the more open space between the particles will be created. This result is confirmed by TGA analysis, which demonstrated the linear relationship between weight loss and fuel content.

#### 3.1.4 TEM analysis

**Fig. 5:**
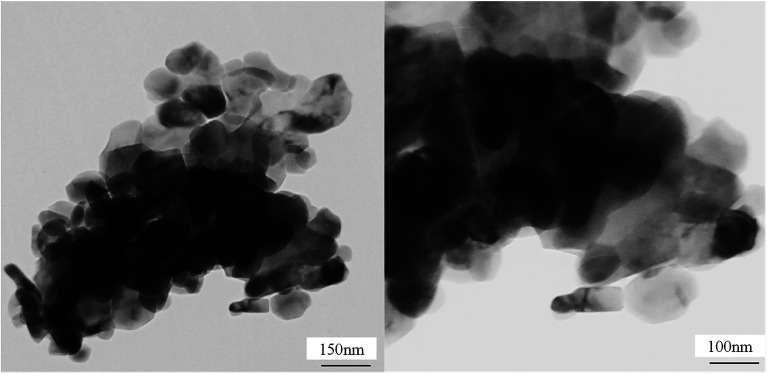
TEM images of NiMo supported on alumina *via* combustion synthesis method with fuel/nitrate = 4.

To address the dispersion of active metals on the alumina surface, TEM analysis was applied. Fig. 5 represents TEM images of the NMACA4 sample synthesized. As the density of active metals is higher than alumina, it scattered higher electron beam fraction. Therefore, its TEM image is darker than alumina particles. According to this figure, well dispersion of metal nanoparticles on alumina surface was observed. It has been reported that the better distribution of Ni and Mo species over the support surface improve their sulfidation ability as well as their catalytic activity.^4,46^ The observed TEM images imply that a reasonable amount of active site is available on the catalyst surface. This phenomena lead to multilayer adsorption, and consequently enhances the catalytic activity of the obtained nanocatalyst.

#### 3.1.5 BET analysis

**Fig. 6:**
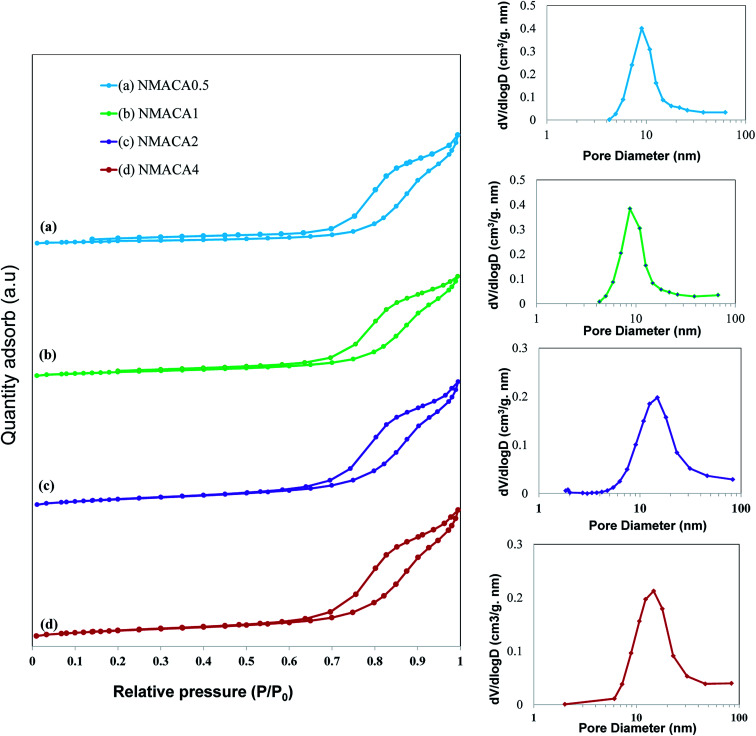
N_2_ adsorption–desorption isotherm of synthesized nanocatalysts with different fuel content.

The nitrogen adsorption–desorption isotherms along with pore diameter distribution of the as-combusted NiMo catalysts are displayed in Fig. 6.

As can be seen in Fig. 6, all the samples show type IV isotherms with H3 hysteresis loop which confirms the slit-shaped mesoporous structure of the catalysts.^47,48^ Little adsorption at low relative pressure (below 0.2) can be attributed to the micropores, while adsorption at relative pressure above 0.7 originated from the presence of mesopores.^35^ A similar result was obtained by Obeso-Estrella *et al.*^49^ Furthermore, the pore diameter distribution of the samples resulted from Barrett–Joyner–Halenda (BJH) calculation indicated narrow distributions at low fuel content. By increasing the fuel content, a shift to larger pore diameters has obtained in the case of NMACA2 and NMACA4, yielding a broaden pore diameter distribution. This result evidences the existence of remarkable amount of mesopores which made the prepared samples suitable for the reactions containing large molecules. This phenomenon is well in agreement with Yousefi *et al.*^48^ report, who synthesized MgO/MgAl_2_O_4_*via* microwave combustion method using various fuels.

In order to better understanding the textural characteristics of the support and NMACA-*x* catalysts, the porosity properties including the mesoporous volume (*V*_meso_), total pore volume (*V*_t_), external surface area (*S*_ext_) and the BET surface area (*S*_BET_) are summarized in Table 1. According to this table, the BET surface area and the total pore volume of the alumina sample are 56 m^2^ g ^−1^ and 0.182 cm^3^ g^−1^, respectively. The BET surface areas of the as-combusted NMACA-*x* series enhanced in the order shown: NMACA0.5 (25 m^2^ g^−1^) < NMACA1 (27 m^2^ g^−1^) < NMACA2 (37 m^2^ g^−1^) < NMACA4 (38 m^2^ g^−1^). It is widely known that the higher surface area facilitates catalytic reactions by affecting the adsorption of the reactant molecules on the metal active phases. By comparing the textural properties of the support and NMACA-*x* catalysts, it can be concluded that NMACA-*x* catalysts have remarkably smaller textural properties than the corresponding support. This observation pointed out some of the support pores were blocked during the deposition of metal species. This observation is well in consistent with our previous research,^50^ where a series of CuO–ZnO–Al_2_O_3_/ZSM-5 catalysts were fabricated *via* solution combustion approach and used in the dimethyl ether production reaction. As listed in Table 1, the external surface areas of the NMACA0.5, NMACA1, NMACA2 and NMACA4 are 14, 22, 33 and 31 m^2^ g^−1^, respectively. Results revealed that the fuel content has an outstanding impact on the porous properties of the catalysts. The mesopore volume of the NMACA0.5, NMACA1, NMACA2 and NMACA4 is equal to 0.096, 0.101, 0.117 and 0.120 cm^3^ g^−1^, respectively. The positive effect of employing higher fuel to oxidant ratio on the textural properties is explained by the higher amount of released gases during the combustion process as evidenced by FESEM analysis.

**Table 1:** Structural properties of nanocatalysts synthesized *via* combustion method using different fuel content

	*S*_BET_ (m^2^ g^−1^)	*S*_ext_ a (m^2^ g^−1^)	*V*_t_ b (cm^3^ g^−1^)	*V*_meso_ c (cm^3^ g^−1^)
Al_2_O_3_	56.7	50.8	0.184	0.182
NMACA0.5	25.9	14.9	0.098	0.096
NMACA1	27.4	22.8	0.103	0.101
NMACA2	37.7	33.1	0.119	0.117
NMACA4	38.1	31.9	0.122	0.120

a *t*-Plot external surface area.

b Volume adsorbed at *P*/*P*_0_ = 0.99.

c Mesopore volume (*V*_total_ − *V*_micro_).

#### 3.1.6 TPR analysis

**Fig. 7:**
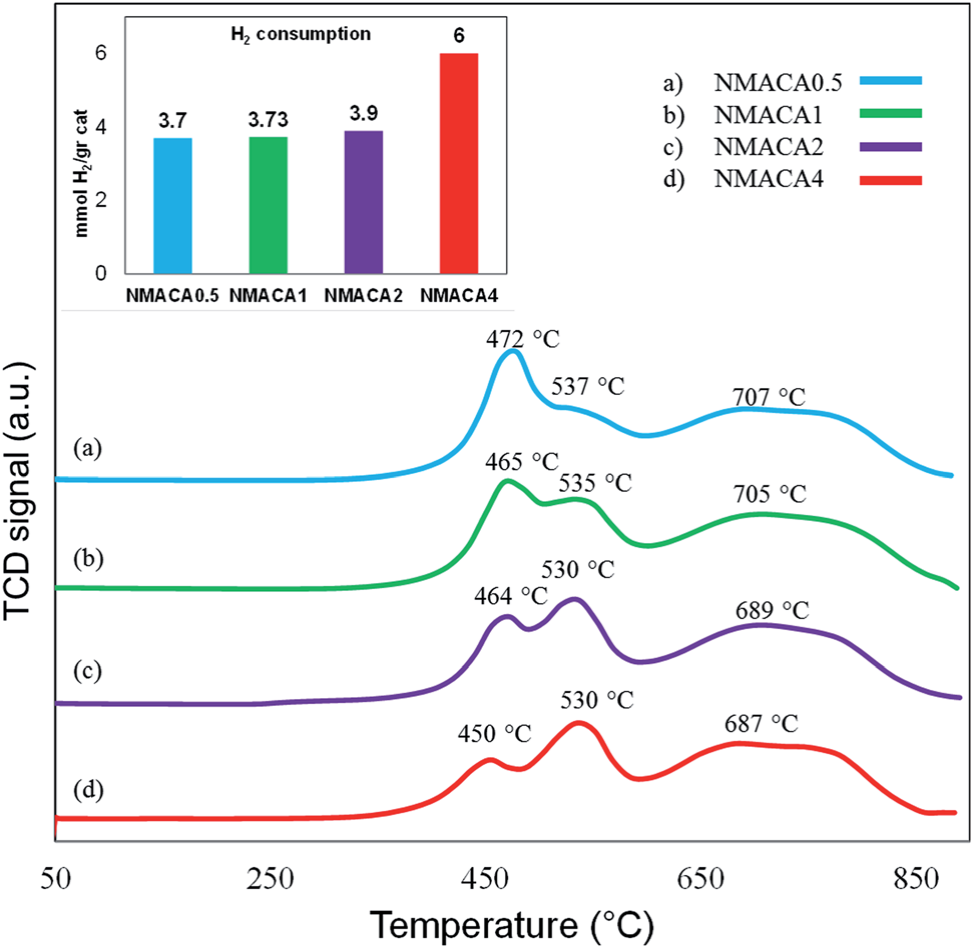
TPR analysis of nanocatalysts synthesized *via* combustion method.

To study the interaction between metal to the support and also reducibility potential of the samples, temperature-programmed reduction analysis was performed. Fig. 7 shows the H_2_-TPR profiles of the NMACA-*x* catalysts. Typically, the TPR patterns display two reduction regions at various temperatures. The first peak located at the temperature range of 300 to 700 °C is attributed to the initial step of octahedral molybdenum reduction (Mo^6+^ → Mo^4+^) which has weak interaction with support.^17^ The second reduction peak between 700 and 1000 °C is assigned to the further reduction of octahedral Mo (Mo^4+^ → Mo^0^) as well as reduction of tetrahedral molybdenum oxide species which have a strong bond to the support. Furthermore, a less intense peak between 600–700 °C has been related to the reduction of Ni^2+^ on the surface of the support.^51,52^ According to a model presented by Topsøe *et al.*^53^ two types of active phases are generated through the sulfidation of HDS catalysts. The structure of type-II is multilayer and is much more active in comparison to the monolayer type-I. The type-II active phase is produced at lower temperature reduction peak. It is worth mentioning that the lower the reduction peak addressed the weaker metal–support interaction. Therefore, the transformation of Mo species from oxide phase to sulfide phase were facilitated.^9^ Considering the H_2_-TPR results, with increasing the fuel content, the position of the first peak shifted to a lower temperature (from 472 °C to 450 °C) which demonstrates weaker interaction of MoO_3_ species with the support and better accessibility of active sites.^9^ This is supported by TEM images of NMACA4 revealing well dispersion of NiMo on the alumina. Furthermore, going from NMACA0.5 to NMACA2, the second peak becomes more intense and a slight shift toward lower temperature has occurred. This phenomenon is more pronounced by evaluating the H_2_ consumption of the NMACA4. The amount of peak area, which arises from adsorbed H_2_ on the catalysts, for NMACA0.5, NMACA1, NMACA2 and NMACA4 were estimated 3.7, 3.7, 3.9 and 6 mmol g^−1^, respectively. Based on these results, the total H_2_ consumption increased by enhancing the citric acid content utilized in the synthesis procedure.

Consequently, the increase of fuel content was beneficial to adjusting the metal–support interactions, which can improve hydrogenation reaction as stated by the previous studies.^16,54^

### 3.2 Comparative performance of nanocatalysts toward HDS reaction

#### 3.2.1 Effect of temperature and time

**Fig. 8:**
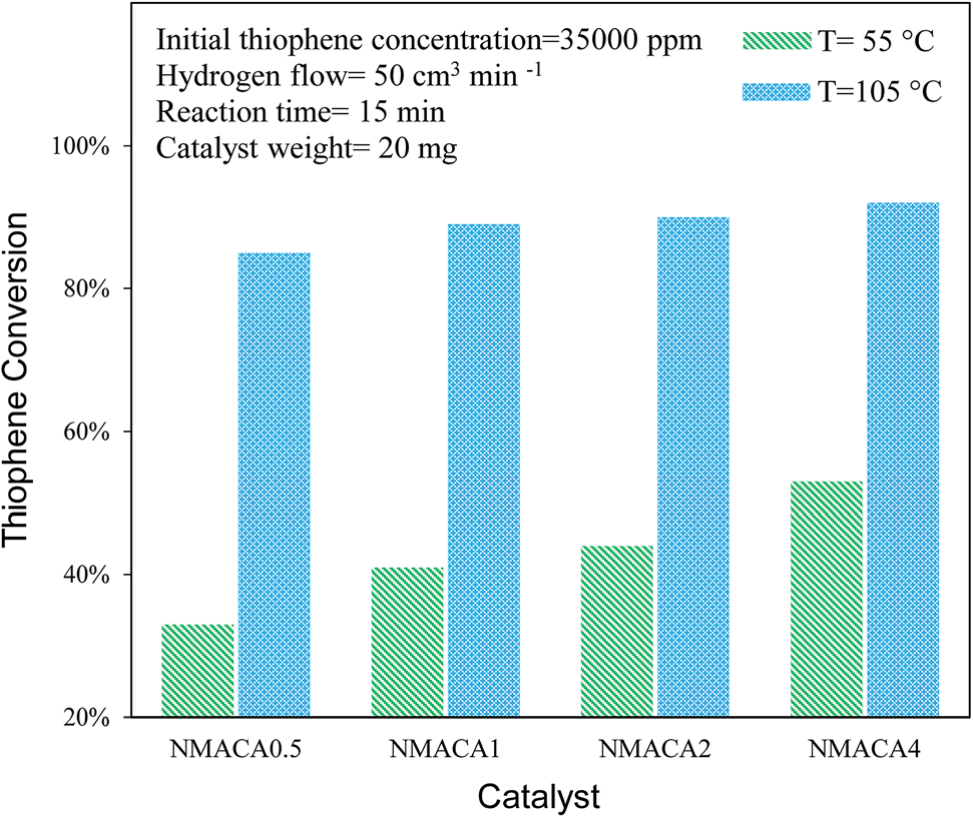
Catalytic activity of NiMo/Al_2_O_3_ with various fuel content toward hydrodesulfurization of thiophene at 15 min.

**Fig. 9:**
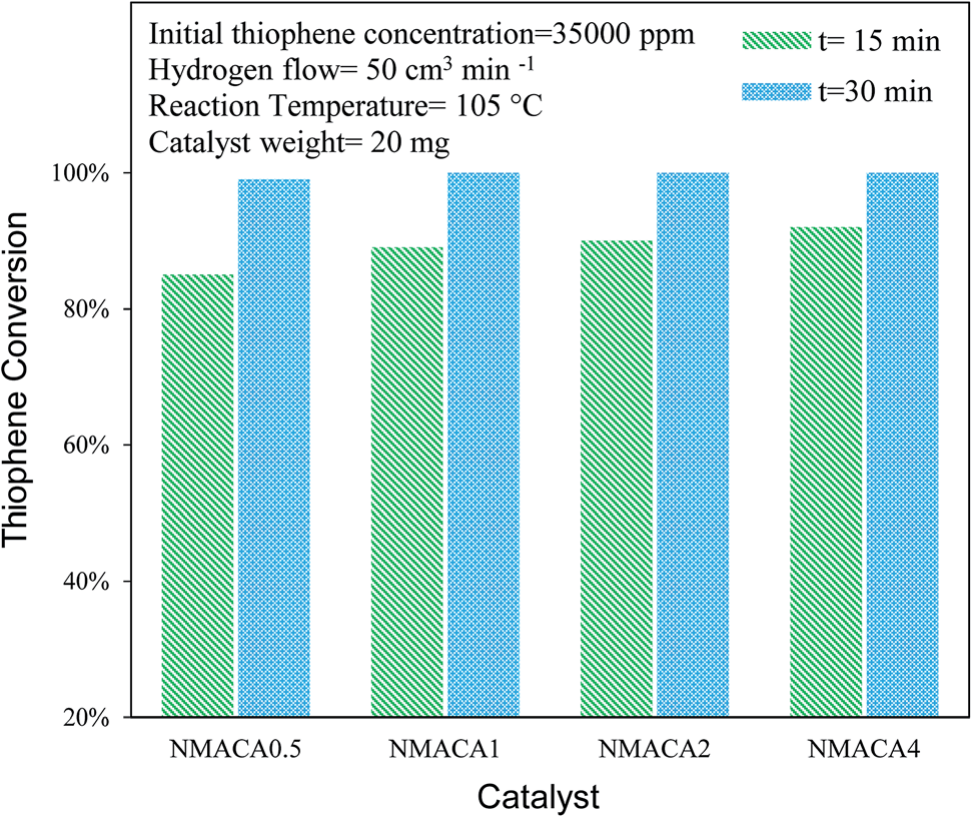
Catalytic activity of NiMo/Al_2_O_3_ with various fuel content toward hydrodesulfurization of thiophene at 105 °C.

The performance of the sulfided catalysts was assessed in the HDS of the thiophene (3.5 wt%) performed in a batch reactor under atmospheric pressure at two temperatures (55 and 105 °C) and reaction times (15 and 30 min). In Fig. 8, the thiophene conversion of the four samples as a function of temperature at 15 min is compared. The results revealed that the hydrodesulfurization of thiophene is sensitive to the temperature which is in line with the previous study.^4^ According to Fig. 8, the thiophene removal at 55 °C for NMACA0.5, NMACA1, NMACA2 and NMACA4 samples are 33%, 41%, 44% and 53%, respectively. Going temperature from 55 °C to 105 °C the amount of thiophene conversion for NMACA0.5, NMACA1, NMACA2 and NMACA4 has obtained 85%, 89%, 90% and 92%, respectively. The results imply that at low temperature the effect of catalyst type is more tangible than high temperature. As seen, the best catalytic activity is obtained at 105 °C for NMACA4, which demonstrated the activity of catalyst increases with increasing the reaction temperature as well as fuel content. A similar trend was reported by Lai *et al.*^41^ The better HDS activity of the NMACA4 sample compared with other samples suggests that the suitable structure of the mentioned catalyst performs a positive role in decreasing the diffusion resistance and mass transfer limitation. This result is consistent with results obtained by Huang *et al.*,^8^ who reported that the porous structure of Co–Mo–Ni/γ-Al_2_O_3_ demonstrated much better hydrodesulfurization performance. Moreover, the superior performance of NMACA4 might be attributed to the highly porous morphology, which is supported by FESEM and N_2_ adsorption–desorption analyses. Also, the ease of reducibility of the NMACA4 compared to the other three catalysts as seen in the H_2_-TPR curve (Fig. 7) is another determining factor for its superior catalytic activity. Based on Topsøe *et al.*^53^ work, the increment of the HDS activity over NiMo/Al_2_O_3_ catalyst is related to the formation of Ni–Mo–S active site on the edge of MoS_2_ slabs, which assist the direct desulfurization of thiophene.^45^Fig. 9 displays thiophene conversion over the synthesized NMACA-*x* series catalysts with two reaction times (15 and 30 min) at 105 °C. Clearly, thiophene conversions enhanced with increasing reaction time Accessibility of the reactants to active site has been increased with prolonging reaction time. Therefore, the diffusion resistance of reactants and products decreased. According to Fig. 9, complete conversion has been obtained for NMACA1, NMACA2 and NMACA4 catalysts after 30 min. Table 2 provides a comparison of the catalytic activity results in the current work with the ones reported in the literature. The observed catalytic activity of the samples prepared in this study exhibits a suitable activity in comparison to the reported results in the literature. Beside the excellent performance of the prepared catalysts in this study, they were prepared by a far simpler and faster procedure than all the aforesaid catalysts. This issue makes them a suitable heterogeneous catalyst for hydrodesulfurization process.

**Table 2:** Comparing the catalytic activity of this study and other reported earlier

Catalysts	Reactor type	Sulfur concentration (ppm)	Sulfur compound	Reaction conditions	Conversion	Ref.
CoMoW/Al_2_O_3_–TiO_2_	Batch	2260	Thiophene	*T* = 320 °C	85%	49
				*P* = 55 atm		
NiMo/Al_2_O_3_	Fixed bed	5000	Dibezothiophene	*T* = 300 °C	95%	9
				*P* = 30 atm		
Co–Mo–Ni/TiO_2_–Al_2_O_3_	Fixed bed	1500	Thiophene	*T* = 74 °C	80%	60
				*P* = 35 atm		
AlCNFMoCo	Batch	550	Dibezothiophene	*T* = 300 °C	99%	17
				*P* = 55 atm		
NiMoW/Al_2_O_3_	Batch	1300	4,6-Dimethyldibenzothiophene	*T* = 300 °C	69%	5
				*P* = 73 atm		
NiMo/Al_2_O_3_–ZrO_2_	Batch	10 000	Thiophene	*T* = 160 °C	99%	18
				*P* = 1 atm		
NiMoW/FMWCNT	Batch	10 000	Thiophene	*T* = 160 °C	100%	27
				*P* = 1 atm		
AlMoCoB5%	Batch	650	Dibezothiophene	*T* = 300 °C	98%	61
				*P* = 50 atm		
FeMoS/Al_2_O_3_ and carbon	Batch	1000	Thiophene	*T* = 280 °C	30%	62
				*P* = 1 atm		
NiMo/Al_2_O_3_	Batch	35 000	Thiophene	*T* = 105 °C	100%	This work
				*P* = 1 atm		

#### 3.2.2 Hydrodesulfurization reaction mechanism

Despite the extensive studies reported so far on the mechanism of thiophene HDS, there is still debate on this reaction mechanism. Wang *et al.*^55^ presented both hydrogenation (HYD) and direct desulfurization (DDS) pathways for thiophene hydrodesulfurization. In the DDS pathway, the thiophene ring is directly broken owing to attack by surface adsorbed hydrogen at the sulfur atom, leaving H_2_S and unsaturated C_4_ hydrocarbons. In the HYD pathway, hydrogenation of thiophene ring occurs before the C–S bond cleavage.^7,56^ The thiophene HDS proceeds first through butadiene intermediate, while butene and butane were formed later. The conversion of butene to butane is very slow compared to the formation of butanes from butadiene.^56^ According to the literature, the mechanism of hydroprocessing reaction is carbonation. Both free radicals and carbonations contribute to that mechanism. Hydrogen atom is able to remove sulfur atom from catalyst surface and create vacancies. Desulfurization starts by adsorption of sulfur atom of the reactant on the sulfur vacancies.^57^ In this regard thiophene (T) and H_2_S adsorb on the sulfur vacancies indicated as Δ. Hydrogen has been subjected to the dissociative adsorption and adsorb on different vacancies denoted as θ:^58^T + Δ ↔ TΔH_2_ + 2θ ↔ 2HθTΔ + 2Hθ → B + SΔ + 2θSΔ + H_2_ → H_2_S + Δ

In the above equations, (B) referred to butadiene which can react further to butenes and butane in kinetically negligible steps. The rate-determining step is considered as the surface reaction between adsorbed hydrogen atom and thiophene with producing butadiene. The hydrogenation of butadiene to butenes and butane is regarded kinetically irrelevant.^56^ The main products for the thiophene HDS are butene and butane which are produced by carbon–sulfur scission of thiophene. Also, 2,3-dihydrothiophene, 2,5-dihydrothiophene and tetrahydrothiophene are important intermediates that can be produced in the reaction mechanism.^59^ It is interesting to note that the obtained products depend on the reaction conditions. For example, tetrahydrothiophene is a major intermediate at low temperature and high pressure, but may not be appeared at atmospheric pressure.^27^

## 4 Conclusions

NiMo/Al_2_O_3_ nanocatalysts were fabricated by the solution combustion method and the effect of various fuel to oxidant molar ratios of 0.5, 1, 2 and 4 were investigated. The formation of MoO_3_ and NiMoO_4_ as the crystalline phase over alumina were confirmed by XRD analysis. According to FESEM and TGA analyses with increasing fuel content, the amount of surface holes and weight loss were enhanced proportionally. The resulted data from nitrogen adsorption–desorption demonstrated that porous properties of the as-combusted catalysts were significantly dependent upon the amount of fuel used. Based on TPR results hydrogen consumption increases with enhancing the fuel/oxidant ratio. Furthermore, the peak position shifted to a relatively lower temperature, indicating more reducible species as well as weakened metal–support interaction. The catalytic activity of prepared samples was evaluated in hydrodesulfurization of thiophene. It was found that the catalysts synthesized with fuel/nitrate = 4 showed higher activity than other three catalysts under moderate operation condition (*T* = 55, 105 °C and *t* = 15 min). Further increase of reaction time from 15 to 30 min led to 100% conversion of thiophene for NMACA1, NMACA2 and NMACA4 samples. The considerable point in the current study was the utilization of a fast, simple and cost-effective approach for the fabrication of the NiMo/Al_2_O_3_ catalysts used in HDS process which represented good performance in harsh operating conditions.

## Conflicts of interest

There are no conflicts to declare.

## Supplementary Material

RA-010-D0RA01590C-s001
