# Supplementary material for: Facile fabrication, characterization and catalytic activity of a NiMo/Al2O3 nanocatalyst via a solution combustion method used in a low temperature hydrodesulfurization process: the effect of fuel to oxidant ratio
Source: RSC Adv. 2020 Mar 26;10(21):12439–50. doi: 10.1039/d0ra01590c (PMC9051088; doi:10.1039/d0ra01590c)
Supplement: RA-010-D0RA01590C-s001 [file RA-010-D0RA01590C-s001.pdf]

Electronic Supplementary Material (ESI) for RSC Advances.
This journal is © The Royal Society of Chemistry 2020

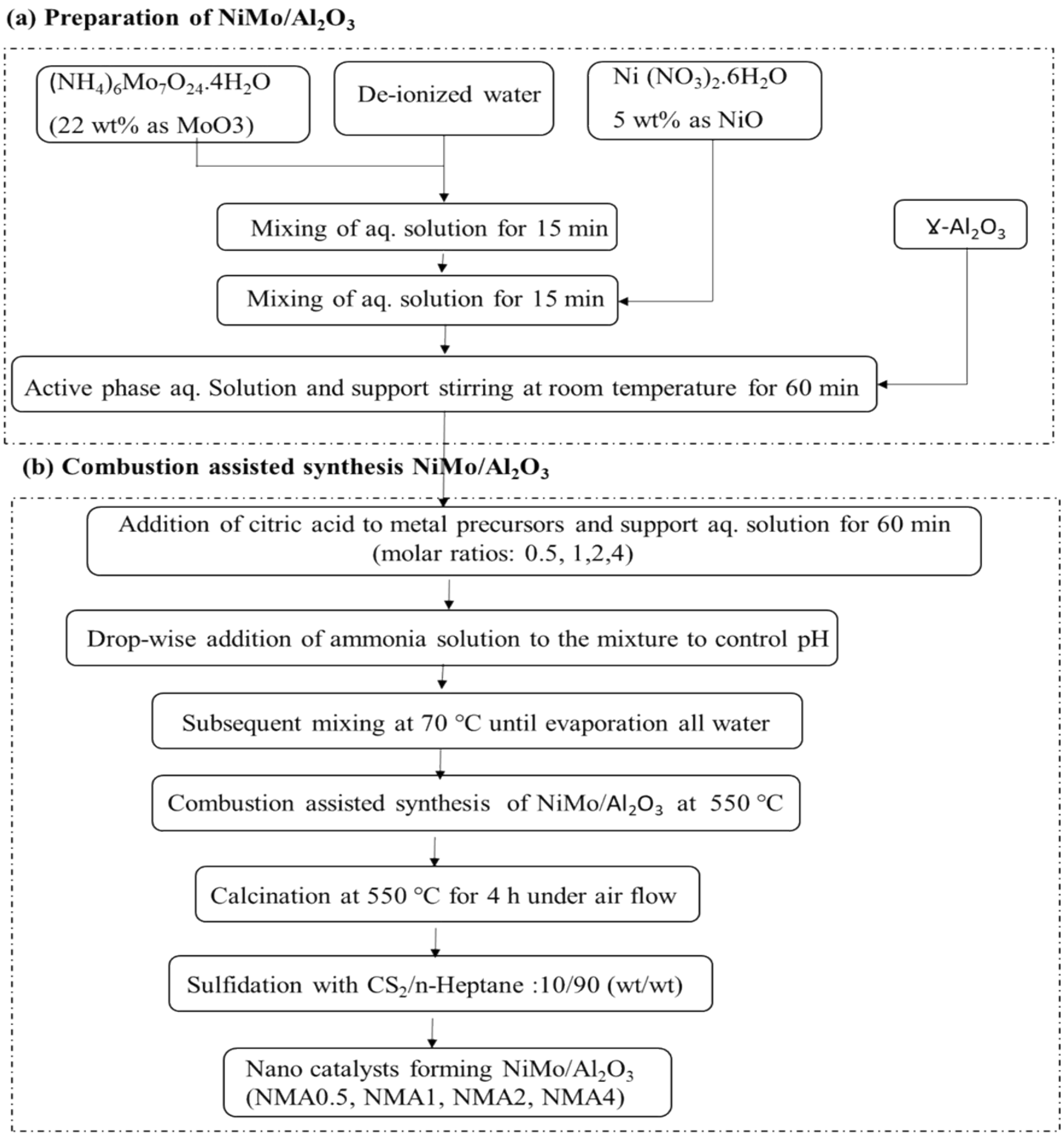


**Figure 1.S. Preparation steps of combustion assisted synthesis of $NiMo/Al_2O_3$**

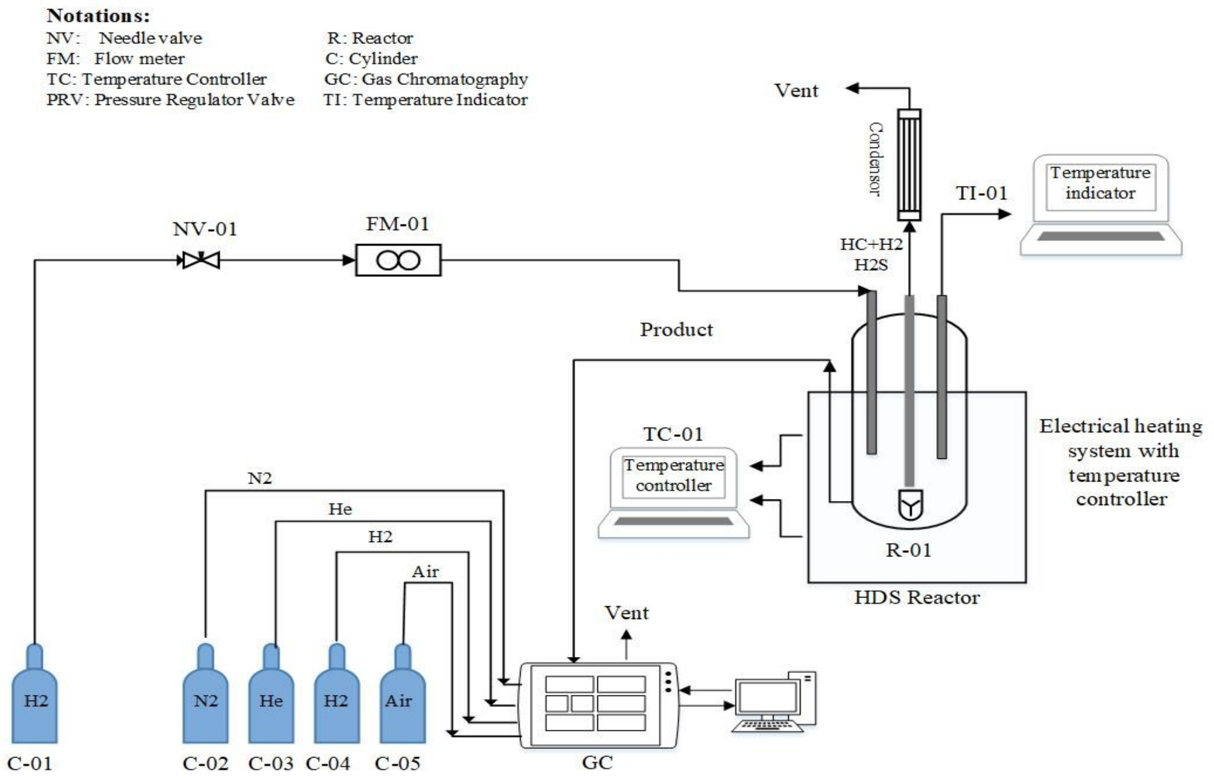


**Figure 2.S. Schematic diagram of experimental setup toward hydrodesulfurization process over combusted NMACA-x nanocatalysts**
